# Ensemble downscaling in coupled solar wind-magnetosphere modeling for space weather forecasting

**DOI:** 10.1002/2014SW001064

**Published:** 2014-06-09

**Authors:** M J Owens, T S Horbury, R T Wicks, S L McGregor, N P Savani, M Xiong

**Affiliations:** 1 Space Environment Physics Group, Department of Meteorology, University of ReadingReading, UK; 2 Space and Atmospheric Physics, Imperial College LondonLondon, UK; 3 NASA Goddard Space Flight CenterGreenbelt, Maryland, USA; 4 Astronomy Department, University of MarylandCollege Park, Maryland, USA; 5 Department of Physics and Astronomy, Dartmouth CollegeHanover, New Hampshire, USA; 6 School of Physics, Astronomy, Computational Sciences, George Mason UniversityFairfax, Virginia, USA; 7 State Key Laboratory for SpaceWeather, Center for Space Science and Applied Research, Chinese Academy of SciencesBeijing, China

**Keywords:** space weather, numerical modelling, stochastic processes

## Abstract

Advanced forecasting of space weather requires simulation of the whole Sun-to-Earth system, which necessitates driving magnetospheric models with the outputs from solar wind models. This presents a fundamental difficulty, as the magnetosphere is sensitive to both large-scale solar wind structures, which can be captured by solar wind models, and small-scale solar wind “noise,” which is far below typical solar wind model resolution and results primarily from stochastic processes. Following similar approaches in terrestrial climate modeling, we propose statistical “downscaling” of solar wind model results prior to their use as input to a magnetospheric model. As magnetospheric response can be highly nonlinear, this is preferable to downscaling the results of magnetospheric modeling. To demonstrate the benefit of this approach, we first approximate solar wind model output by smoothing solar wind observations with an 8 h filter, then add small-scale structure back in through the addition of random noise with the observed spectral characteristics. Here we use a very simple parameterization of noise based upon the observed probability distribution functions of solar wind parameters, but more sophisticated methods will be developed in the future. An ensemble of results from the simple downscaling scheme are tested using a model-independent method and shown to add value to the magnetospheric forecast, both improving the best estimate and quantifying the uncertainty. We suggest a number of features desirable in an operational solar wind downscaling scheme.

**Key Points:**

- Solar wind models must be downscaled in order to drive magnetospheric models
- Ensemble downscaling is more effective than deterministic downscaling
- The magnetosphere responds nonlinearly to small-scale solar wind fluctuations

## 1. Introduction

Increasing reliance on space-based technologies, notably GPS and continuous satellite-based Earth observation, leads to increasing societal need to characterize and forecast space weather, particularly geomagnetic storms. These are periods of enhanced disturbances to the terrestrial magnetosphere resulting from variability in the near-Earth solar wind [e.g., *Feynman and Gabriel*, 2000; *Hapgood*, 2011]. Such storms pose a serious threat to the power grid via geomagnetically induced currents, particularly as the system is restructured to incorporate renewable power sources [e.g., *Thomson et al.*, 2010].

Geomagnetic activity can, in principle, be forecast through magnetospheric and ionospheric simulations driven by upstream solar wind conditions (though there remain many technical and scientific challenges). While ionospheric conductivity is an important and active inner-magnetospheric boundary condition [e.g., *Merkin and Lyon*, 2010], the most common approach to magnetospheric modeling is simply to impose the outer boundary conditions using in situ solar wind observations from spacecraft at the first Lagrange point (L1). As L1 is approximately 99% of the distance from the Sun to the Earth, the solar wind undergoes little further evolution before arriving at the magnetosphere. Thus, L1 is an ideal position for very short lead time or “nowcasting” of magnetospheric and ionospheric conditions, but any forecast lead time is limited to well under an hour. Solar wind observations from closer to the Sun could, theoretically, extend this lead time significantly. Maintaining a spacecraft in such an orbit, however, is difficult and no such capability currently exists.

**Figure 1:**
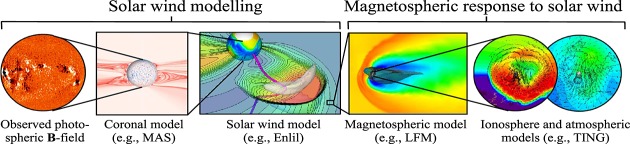
A coupled numerical scheme for predicting space weather using photospheric magnetic field observations. Using model solar wind output to drive magnetospheric simulations requires significant spatial and temporal downscaling of the time series.

By driving magnetospheric simulations with predictions from coronal and solar wind models, as opposed to L1 spacecraft observations, the forecast lead time can be extended from under an hour to 2–4 days, the solar wind travel time from the Sun to Earth. Figure 1 shows a chain of coupled numerical models for space weather forecasting, ultimately driven by remotely sensed photospheric magnetic field observations, though white light observations of the corona are also being investigated as initialization method. Individual model components are those used in *Luhmann et al.* [2004] and *Merkin et al.* [2007], listed here only as examples. Such “Sun-to-Earth” forecasting of geomagnetic storms can be broadly considered a two-step process: forecasting the solar wind conditions in near-Earth space, and forecasting the subsequent magnetospheric response to those solar wind conditions.

**Figure 2:**
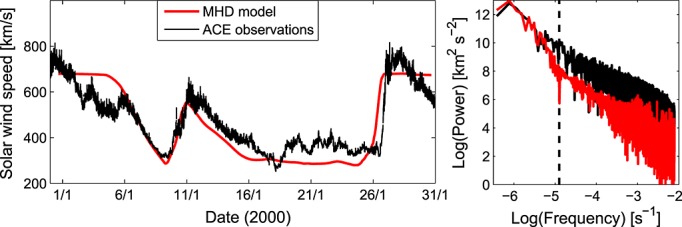
(left) The solar wind speed in near-Earth space for Carrington Rotation 1958 (i.e., January 2000). Black: 64 s resolution observations from the Advanced Composition Explorer (ACE) spacecraft. Red: Model predictions based upon Kitt Peak magnetograms. (right) The power spectrum in the same format. Although the large-scale structure of the solar wind is very well reproduced by the numerical modeling scheme during this particular interval, fluctuations below approximately 1 day (shown as the vertical dashed line) are much weaker. This is a fundamental limitation of using magnetogram-derived solar wind properties to drive magnetospheric simulations.

As discussed, solar wind models are initiated with coronal models which are constrained by photospheric magnetic field data. This approach is often capable of reproducing the steady state, large-scale structure of the ambient solar wind in near-Earth space [e.g., *Owens et al.*, 2008] (see also Figure 2), though there is obviously much ongoing work to further improve their predictive capability. Initial attempts at simulating large-scale transient structures such as coronal mass ejections are also promising [*Titov et al.*, 2008]. However, using solar wind model output to drive magnetospheric models presents a fundamental problem: the magnetosphere is sensitive to both the large-scale structure, which is captured by solar wind models, and small-scale fluctuations which are far below both typical solar wind model spatial and temporal scales [*Borovsky and Funsten*, 2003; *Merkin et al.*, 2007]. Much of this solar wind “noise,” loosely defined here as fluctuations below the 1 day time scale (shown as the vertical dashed line in Figure 2), is likely the result of stochastic processes such as turbulence [e.g., *Horbury et al.*, 2001; *Alexandrova et al.*, 2009]. Thus, even substantial improvements/developments in the numerics and physics of solar wind models are unlikely to be able to deterministically forecast these structures. As the solar wind noise can change simulated magnetospheric responses by an order of magnitude [*Merkin et al.*, 2007], a qualitatively different approach is required.

## 2. Downscaling Techniques

The terrestrial weather- and climate-modeling communities have addressed similar scaling issues to those described in the previous section. For example, global climate models (GCMs) are used to predict precipitation, which is typically determined by processes on spatial scales finer than GCMs can resolve [*Christensen and Christensen*, 2003]. Two different approaches are generally taken [e.g., *Christensen and Christensen*, 2003; *Maraun et al.*, 2010, and references therein]. The first method is to nest higher spatial resolution grids within coarser global models, allowing finer-scale structure to be resolved where required. This method would not allow deterministic forecast of solar wind noise but could, in principle, predict the correct spectral characteristics. However, as there is evidence of small-scale structure convecting from the Sun to Earth [*Viall et al.*, 2009], this would require higher spatial resolution along the entire Sun-Earth passage, which would be both computationally expensive and highly model dependent, requiring individual implementation in whatever solar wind code is used. Furthermore, it is not clear that even with increased resolution current solar wind models would be able to accurately capture physical processes responsible for turbulent formation and evolution [e.g., *Bruno and Carbone*, 2005, and references therein]. The second method is to use statistical relations between large- and small-scale parameters. If it is assumed that a model perfectly reproduces the large-scale parameters (i.e., it provides a “perfect prognosis” at these time scales), the downscaling can be based entirely on regression between observed small-scale and observed large-scale parameters, as the latter are assumed to be identical to the model outputs. While this will not correct for any systematic biases in the model-predicted solar wind, it is entirely model independent. Conditional weather generators perform a similar function, but instead of using large-scale parameters to make a single small-scale prediction, they generate a randomized time series at the local scale with the correct spectral properties [*Wilks and Wilby*, 1999].

Previous studies suggest that magnetospheric response to small-scale solar wind structure is primarily determined by the spectral properties of the fluctuations rather than their precise timing and phasing. The inclusion of the solar wind fluctuations increases the dayside magnetospheric ULF wave power [*Huang et al.*, 2010] and consequently improves statistical agreement with observations of the inner magnetosphere during high-speed solar wind streams (S. L. McGregor et al., Modeling magnetospheric response to synthetic Alfvénic fluctuations in the solar wind: 2. ULF wave fields in the magnetosphere, submitted to *Journal of Geophysical Research*, 2014b). However, the magnetosphere is such an integrated and stochastic system, that simulations can only predict statistical patterns of plasmoid response (S. L. McGregor et al., Modeling magnetospheric response to synthetic Alfvénic fluctuations in the solar wind: 1. Effects on plasmoid evolution, submitted to *Journal of Geophysical Research*, 2014a). Thus, a “perfect prognosis weather generator,” which assumes the undownscaled model solar wind values are correct and simply adds appropriate noise, is a valid approach to the space weather scaling issue. We note that downscaling the magnetospheric, rather than solar wind, model results would be more computationally efficient, but the non-linear response of the magnetosphere to small-scale structure in the solar wind renders this approach inadequate. In the remainder of this paper, we put together a simple version of a solar wind downscaling scheme and demonstrate both how best to validate the process and the value it adds to space weather forecasting.

## 3. Introducing Noise to Solar Wind Models

**Figure 3:**
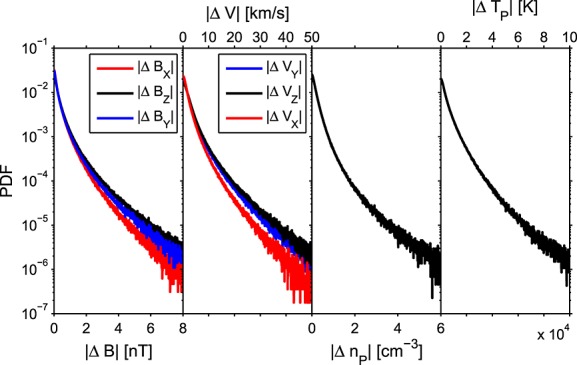
Probability distribution functions of Δ*X*, point-to-point differences in various solar wind parameters.

**Figure 4:**
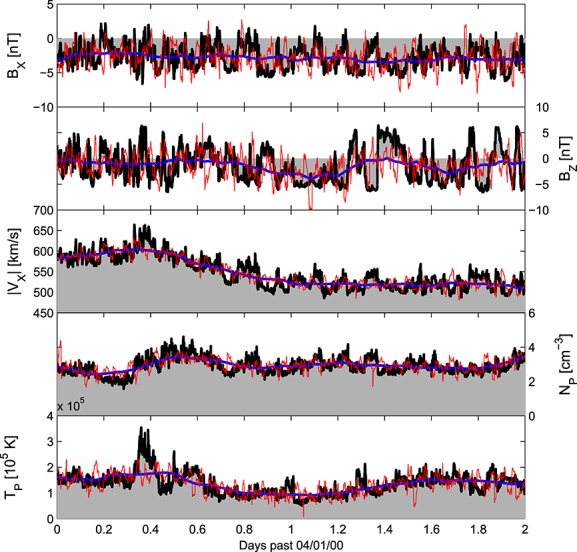
The 2 day solar wind interval used in this study. The observed 64 s ACE solar wind time series is shown in black and as grey-shaded regions, while the model-like time series, obtained from an 8 hour filter of the ACE data, is shown in blue. The downscaled model-like series is shown in red. (top to bottom) The radial (*B*_*X*_) and out-of-ecliptic (*B*_*Z*_) components of the magnetic field in GSE coordinates, followed by the radial solar wind speed, |*V*_*X*_|, the proton density, *n*_*P*_, and the proton temperature, *T*_*P*_.

The Advanced Composition Explorer (ACE) spacecraft provides continual in situ measurements of the solar wind in near-Earth space. The entire ACE magnetic field and plasma data set (1998–2011) at 64 s resolution, the spin period of the spacecraft, is used to produce probability distribution functions (PDFs) of Δ*X*, point-to-point changes in solar wind parameter *X* (as per *Borovsky* [2008] and *Owens et al.* [2011]), in the three components of magnetic field vector, three components of the proton velocity vector, proton density, and temperature. These are shown in Figure 3. From the PDFs, we generate cumulative distribution functions (CDFs). These are used to introduce high-frequency noise to solar wind model time series. To demonstrate and test this process, we select a 2 day interval from 4 January 2000 to 6 January 2000. Two days is chosen as it is a reasonable run time for a magnetospheric simulation. The black line in Figure 4 shows that this interval is rather unremarkable in character though does feature both fast and slow solar wind.

The first step is to produce a “model-like” time series for this interval, which perfectly reproduces the large-scale features, but none of the noise. The simplest method is to smooth the observed ACE time series with an 8 h filter. The result is shown as the blue lines in Figure 4. This level of filtering produces a similar “smoothness” in the time series to current solar wind models [*Riley et al.*, 2001; *Odstrcil et al.*, 2004; *Tóth et al.*, 2005]. Using smoothed observations, rather than actual model data, allows testing of the downscaling scheme without introducing any solar wind model bias. This synthetic model-like time series correctly reproduces the large-scale features of the solar wind but does not capture the small-scale fluctuations.

The next step is to downscale the model-like time series. The process described here is the simplest means to test the general downscaling approach, rather than an operationally ready downscaling scheme. We use a random number generator (RNG) to create a series of numbers between 0 and 1 with uniform probability. These are used to draw values of |Δ*X*|from the appropriate observationally determined CDF. A second RNG series is used to make approximately half the solar wind changes negative, creating a Δ*X*time series. The *X* noise time series is then assembled by joining successive Δ*X* values. As the large-scale structure is assumed to be captured by the solar wind model, the noise-only time series is linearly detrended in 1 h chunks to remove any drifts from the RNG (however, as fluctuations are approximately symmetric about zero, very little detrending is actually required and only weak 1 hour periodicity is introduced). Finally, this noise time series is added to the model-like time series to give the downscaled model-like time series, shown as the red line in Figure 4. As expected, the downscaled model-like time series shows the same large-scale trends as the undownscaled model-like series but similar small-scale variability to the ACE solar wind. Obviously, the downscaled noise does not provide a one-to-one match with the observed fluctuations, but as a RNG was used, this is only one possible solution and an ensemble of downscaled solar wind time series can easily be produced (see section 5). Note also that the largest observed changes in, e.g., *B*_*Z*_, are not present in the downscaled time series as the simple PDF approach does not include the effect of intermittency across time scales other than 64 s, etc.

## 4. Magnetospheric Response

The “skill” gained by the using the downscaling scheme, if any, is now tested by comparing the magnetospheric response with and without the downscaling scheme. We use the Lyon-Fedder-Mobbary (LFM) [*Lyon et al.*, 2004] magnetospheric/ionospheric simulation, version 2.1.1, at NASA's Community Coordinated Modeling Center (CCMC). Both the downscaling scheme itself and testing procedure are required to be independent of both solar wind and magnetospheric models, as many different combinations of models are possible. While we must necessarily assume that the magnetospheric model being used responds to small-scale solar wind changes in the correct sense, the following approach is taken in order to minimize any further magnetospheric model bias in validation:

1. LFM is first run using the actual 64 s ACE observations, providing a “baseline” result against which all other runs are compared. Any systematic bias in the magnetospheric model is incorporated at this stage.
2. LFM is run using the undownscaled model-like time series (i.e., ACE observations with 8 h filter). The difference between this result and the baseline will be entirely due to the lack of solar wind noise as the same magnetospheric model bias is present in both sets of results.
3. LFM is then run a third time using the downscaled model-like time series. Comparison of this result with both the baseline and undownscaled model-like results quantifies any skill added by the downscaling scheme.

**Figure 5:**
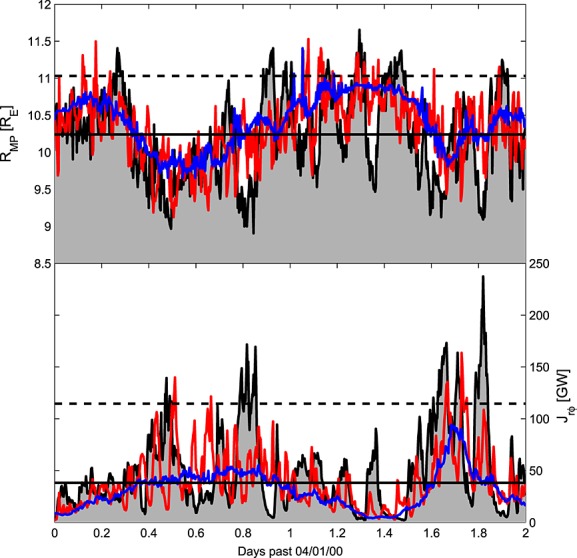
Magnetospheric response to different solar wind time series. Black, blue, and red lines show the observed, undownscaled, and downscaled model-like time series, respectively. (top) The magnetopause standoff distance at local noon (*R*_MP_). (bottom) Ionospheric Joule heating computed from radial current and electric potential (*J*_*Rφ*_. Solid (dashed) horizontal lines show the observed 50th (90th) percentile values.

This process could be applied to any metric of magnetospheric disturbance, ideally chosen to specifically emphasize the desired forecasting application. In this study we use two simple diagnostics of the global magnetospheric state, which are routinely calculated as part of the CCMC's simulation runs, namely, the magnetopause standoff distance at local noon, *R*_MP_, and ionospheric Joule heating computed from radial current and electric potential, *J*_*Rφ*_. For the purposes of demonstrating the downscaling validation process, the details of these properties are somewhat irrelevant; what is important is how these parameters compare between runs using the observed solar wind, undownscaled model-like, and downscaled model-like time series, shown as black, blue, and red lines in Figure 5, respectively.

Table1 lists the statistical properties of the magnetospheric response to the various solar wind time series. In general, the effect of this single realization of downscaling is to bring the mean and standard deviations of magnetospheric parameters closer to those obtained from the use of actual ACE observations (this is further illustrated in the reliability diagrams in Figure 7, discussed in the next section). Despite this statistical improvement, this single downscaling instance also increases the point-by-point error (i.e., the mean-square error increases, and the linear correlation decreases). Note, however, that point-by-point analysis may not be the best assessment of the usefulness of a forecast, as it frequently over penalizes forecasts which exhibit the correct variability but contain small timing errors [e.g., *Owens et al.*, 2013, Figure 8]. In the next section, we test the value added to downscaled forecasts using an ensemble approach rather than a single realization.

**Table 1:** Magnetospheric Response to Driving by Various Solar Wind Time Series^a^

	*R*_MP_	(*R*_*E*_)			*J*_*Rφ*_ Giga Watts (GW)			
	Mean	SD	*r*_*L*_	MSE	Mean	SD	*r*_*L*_	MSE
ACE observations	10.23	0.579	–	–	50.1	40.9	–	–
Undownscaled model-like time series	10.41	0.349	0.537	0.490	31.8	18.3	0.530	34.9
Single model-like downscale	10.31	0.440	0.376	0.581	39.8	27.9	0.408	39.0

a SD = standard deviation; MSE = mean-square error.

## 5. Ensemble Results

**Figure 6:**
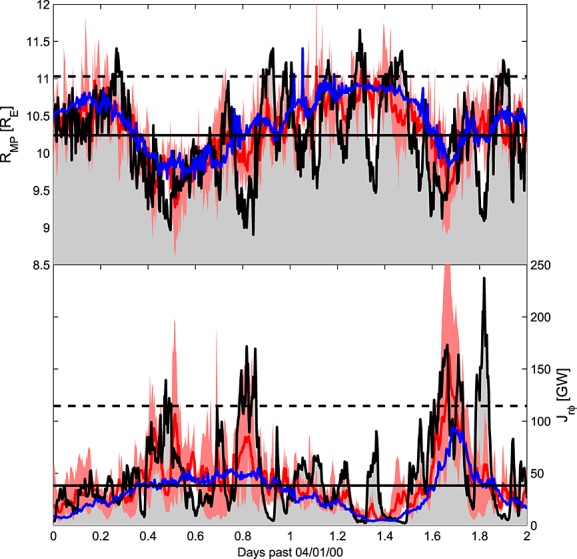
Same as Figure 5 but with the red line showing the downscaled ensemble mean and pink-shaded region showing the spread between maximum and minimum values of ensemble members.

As the noise added by the downscaling scheme is produced by a random number generator, it is trivial to produce multiple noise realizations. By running the magnetospheric model multiple times with each downscaled model-like time series, we can produce a simple ensemble forecast [*Leutbecher and Palmer*, 2008] of magnetospheric conditions. Figure 6 shows the results from an ensemble of five magnetospheric model runs driven by five individual model-like downscales (including the one shown in Figure 5), in a similar format to Figure 5. The red line shows the ensemble mean, while the pink-shaded area shows the spread between the maximum and minimum values of ensemble members. In an operational forecast capacity, more than five ensemble members would ideally be used.

The ensemble results show a number of important points. As downscaled solar wind noise is random fluctuations about the undownscaled values, a purely linear magnetospheric response would result in the magnetospheric ensemble downscaled mean (red) being identical to the undownscaled run (blue), so long as a sufficiently large number of ensemble members are included. For much of the interval, this is a reasonable approximation, but there are clearly times when the ensemble downscaled mean significantly deviates from the undownscaled magnetospheric response, e.g., *J*_*rφ*_ on days 0.4, 0.8, and 1.7. Here the magnetosphere is better reconstructed when solar wind noise is added, regardless of the precise timing and orientation of these fluctuations. This demonstrates the value in downscaling the solar wind model results, rather than simply downscaling the magnetospheric model output, which would be cheaper computationally. A further point of note, again most prominent in *J*_*rφ*_, is that the ensemble spread is generally greatest when the ensemble mean is furthest from the observed value. This suggests that even when downscaling does not necessarily improve the magnetospheric forecast, it does at least provide a useful estimation in the uncertainty in the forecast, as discussed below.

**Figure 7:**
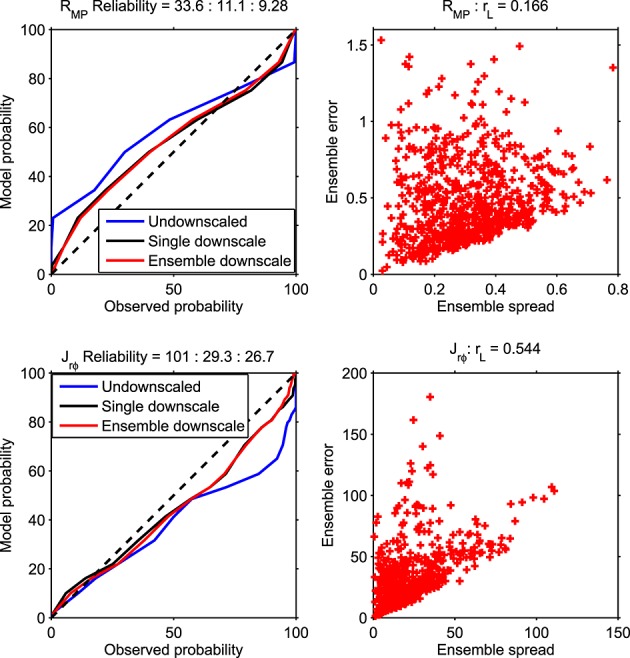
(left) Reliability diagrams for (top) *R*_MP_ and (bottom) *J*_*rφ*_ resulting from undownscaled, single downscale, and ensemble downscale model-like time series. (right) Ensemble spread error diagrams. See text for discussion.

Figure 7 (left) shows magnetospheric reliability diagrams, plots of the observed CDF against that of a forecast, for *R*_MP_ (top) and *J*_*rφ*_ (bottom) resulting from undownscaled, single downscale, and ensemble-downscaled model-like time series. These plots basically quantify a forecast's ability to produce the observed climatology [e.g., *Atger*, 1999]. The mean reliability, *R*, is the average square difference between the observed and forecast frequency in the different CDF categories. Thus, a forecast with a perfect reliability would have *R* = 0, and the associated reliability curve would lie exactly along *y* = *x*, shown by the dashed diagonal line. The undownscaled model-like time series has the worst reliability (33.6 for *R*_MP_ and 101 for *J*_*rφ*_), primarily because its dynamic range does not capture the small values of *R*_MP_ and large values of *J*_*rφ*_ that are observed. The downscaled model-like time series substantially improves the reliability (11.1 for *R*_MP_ and 29.3 for *J*_*rφ*_), though the same basic trends are still present. Even with only five members, the ensemble approach is found to be more a small but significant amount more reliable (9.28 for *R*_MP_ and 26.7 for *J*_*rφ*_) than a single downscaling realization.

Figure 7 (right) shows the “spread” (the standard deviation between ensemble members), as a function of the “error” (the mean-square error between the ensemble mean and observations) [e.g., *Atger*, 1999]. Correlation in the “spread error” plot quantifies how well an ensemble correctly captures periods of uncertainty in the forecast. Both the *R*_MP_ and *J*_*rφ*_ spread error plots show correlation significant above the 95% level (*r*_*L*_ = 0.166 for *R*_MP_ and 0.544 for *J*_*rφ*_). The spread error uncertainty estimate is particularly good for *J*_*rφ*_, which responds highly nonlinearly to solar wind forcing, and suggesting the ensemble approach adds significant value to the forecast.

## 6. Potential Economic Value of Downscaling

The value of a probabilistic forecast ultimately lies in its use as a decision-making tool. Here we use a cost/loss model [*Murphy*, 1977; *Richardson*, 2000], widely used in testing the value of numerical weather predictions, which aims to quantify the benefit (or otherwise) of taking action based upon a forecast. As this method is not widely used in space weather, we here illustrate the basic principle with a hypothetical example. Let us assume that a satellite operator can put his/her hardware into safe mode to protect against space weather effects, which come in to play when a magnetospheric parameter exceeds a threshold *T*. The cost of temporarily interrupting operations is *C*, whereas (s)he will incur an economic loss *L* if *T* is exceeded, and no protective action is taken. It is given that *L* > *C*, else there is no benefit in ever taking action. In space weather applications, where *L* may represent the complete loss of a spacecraft or the extended interruption to a power grid [e.g., *Kappenman*, 2006], *C*/*L* will generally be low.

In the complete absence of any forecast, it follows that the spacecraft should be always operating (always in safe mode) if the climatological probability of exceeding *T* is less (greater) than *C*/*L*. Thus, the climatological expense over any interval, *E*_*C*_, is then the sum of the costs and losses incurred at each time step. In this study, we do not have access to a full LFM climatology of *R*_MP_ and *J*_*rφ*_, so we simply compute the climatological probability over the same 2 day interval that is tested. Thus, *E*_*C*_ will be lower than in a “real-world” scenario and provides a tougher test for the forecasts.

The undownscaled model-like time series and the single downscaling realization both result in a deterministic forecast of magnetospheric properties, in that the forecast value at time *t*, *F*_*t*_, is uniquely either ≤*T* or >*T*. The total forecast “expense,” *E*_*F*_, of acting upon these forecasts is then simply the sum of the cost/loss at each time step, which can be computed from Table2.

**Table 2:** The Expense Matrix For a Deterministic Forecast, Such as the Undownscaled Model-Like Time Series or a Single Realization of Solar Wind Downscaling^a^

		Forecast	
		*F*_*t*_≤*T*	*F*_*t*_>*T*
Observed	*O*_*t*_≤*T*	0	C
	*O*_*t*_>*T*	L	C

a *O*_*t*_(*F*_*t*_) is the observed (forecast) value at time *t*. *T* is the threshold at which action should be taken. *L*is the economic loss suffered by not taking action if *T* is exceeded, while *C* is the cost of taking action.

For the ensemble forecast, each time step has a probability of exceeding *T* at time *t*, *P*_*t*_, determined by the fraction of ensemble members which exceed *T*. In this case, the operator should take action only if *P*_*t*_>*C*/*L*and *E*_*F*_ can then be computed by considering the costs/losses at each time step, computed from Table3.

**Table 3:** The Expense Matrix For a Probabilistic Forecast, Such as the Ensemble of Downscaled Model-Like Time Series^a^

		Forecast	
		*P*_*t*_≤*C*/*L*	*P*_*t*_>*C*/*L*
Observed	*O*_*t*_≤*T*	0	C
	*O*_*t*_>*T*	L	C

a *O*_*t*_ is the observed value at time *t*. *P*_*t*_ is the ensemble forecast probability that *T*, the threshold at which action should be taken, will be exceeded. *L* is the loss suffered by not taking action if *T* is exceeded, while *C* is the cost of taking action.

The potential economic value, *V*,of a forecast is then given by

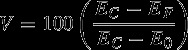
1where *E*_0_ is the minimum expense that could be incurred over the interval considered (i.e., the sum of costs for necessary protective action, which is equivalent to acting on a perfect deterministic forecast). Thus, *V* = 100*%* means the forecast is perfect, whereas *V* = 0*%*means the forecast is more expensive than simple climatology.

**Figure 8:**
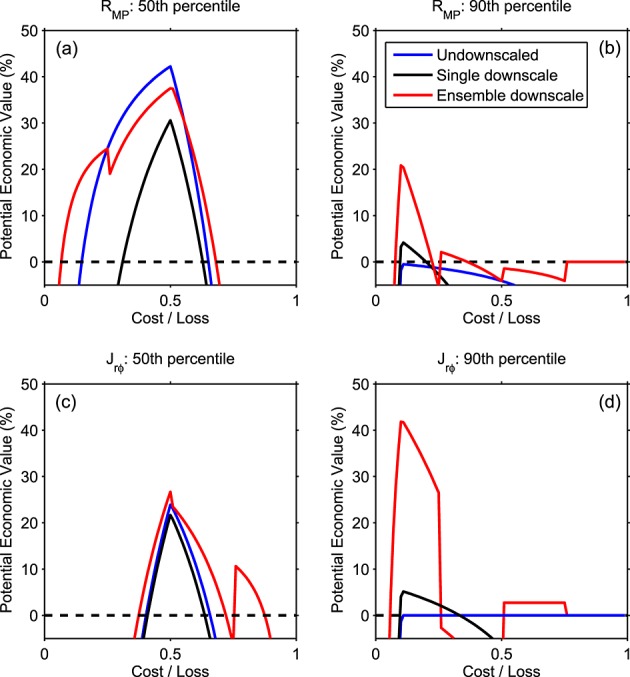
The potential economic value of magnetospheric forecasts driven by different solar wind time series. (top and bottom) The magnetopause standoff distance and ionospheric heating, respectively. (left and right) Action thresholds above the median and 90th percentile. Blue/black/red lines show the undownscaled, single downscale realization, and ensemble downscale, respectively.

Figure 8 shows the potential economic value, *V*, as a function of cost/loss ratio (*C*/*L*), for undownscaled and downscaled driving of the magnetospheric model. We consider two threshold values, the observed 50th and 90th percentiles, equivalent to testing a forecast's ability to predict whether a parameter will be above or below the median, and whether it can predict extremes of the observed distribution, respectively. For the limited intervals under consideration here, Figure 8a shows that the downscaled ensembles improve forecasting relative to the median *R*_MP_ at low and high *C*/*L* but not intermediate values (0.25 < *C*/*L* < 0.55). For extremes of *R*_MP_ (Figure 8b), the undownscaled forecast has no value above climatology at all *C*/*L*values, whereas the downscaled forecast, particularly in ensemble use, shows value at low *C*/*L*. For *J*_*rφ*_, (Figures 8c and 8d) the downscaling ensemble provides a more valuable forecast than the undownscaled solar wind at nearly all *C*/*L*across both thresholds, but again this is most notable for the extreme threshold and at low *C*/*L*.

## 7. Discussion and Conclusions

The first half of this paper outlines a simple method of solar wind downscaling in order to more realistically drive magnetospheric models with the output of solar wind models. It uses the observed solar wind noise characteristics to insert random fluctuations into time series which approximate solar wind models. The second half of the paper demonstrates a model-independent method of testing such downscaling schemes. Applying these tests to the simple downscaling scheme shows that the process adds value to Sun-to-Earth forecasting when an ensemble approach is taken. Downscaling seems to be most beneficial in improving forecasting skill at the extremes of observed conditions, particularly for low cost/loss ratios, which are the most likely parameter regimes in space weather mitigation. There are, however, a number of obvious improvements to the downscaling scheme which will be incorporated in future iterations:

1. There is observational evidence that the spectral properties of solar wind noise vary systematically with solar cycle and between different solar wind types, such as fast and slow solar wind flows [*Yordanova et al.*, 2009; *Borovsky*, 2010; *Hietala et al.*, 2014]. Thus, the solar wind noise PDF should be separated into different solar wind types, namely, fast, slow, and transient/CME solar wind. The relevant noise PDF would then be used to introduce noise into the solar wind model time series.
2. Within different solar wind types, the relation between observed noise and observed bulk solar wind parameters could be quantified in order to generate a multidimensional “lookup” table of noise properties. This should be used to produce a “conditional weather generator,” which further tailors the spectrum of noise introduced into the large-scale properties set by the model time series.
3. Fluctuations in solar wind parameters are known to be coupled, e.g., B and V variations in the presence of Alfvén waves. The coupling between solar wind parameter fluctuations should be quantified and used to produce a more physically realistic noise profile.

Finally, note that the methods of assessment used in this study consider each point in the time series independently. So forecasts with the correct variability but small systematic timing errors will be penalized more severely than a similarly flawed forecast which also underestimates the variability. Thus, if the general form and magnitude of magnetospheric variations are of more value to an operator than the exact timings [e.g., *Kappenman*, 2006; *Thomson et al.*, 2010], an event-based forecast assessment method [e.g., *Owens et al.*, 2005] may be more appropriate. This will form part of a future study.
